# Testosterone in COVID-19: An Adversary Bane or Comrade Boon

**DOI:** 10.3389/fcimb.2021.666987

**Published:** 2021-09-08

**Authors:** Hayder M. Al-kuraishy, Ali I. Al-Gareeb, Hani Faidah, Athanasios Alexiou, Gaber El-Saber Batiha

**Affiliations:** ^1^Department of Clinical Pharmacology and Medicine, College of Medicine, ALmustansiriyia University, Baghdad, Iraq; ^2^Faculty of Medicine, Umm Al Qura University, Mecca, Saudi Arabia; ^3^Department of Science and Engineering, Novel Global Community Educational Foundation, Hebersham, NSW, Australia; ^4^AFNP Med Austria, Wien, Austria; ^5^Department of Pharmacology and Therapeutics, Faculty of Veterinary Medicine, Damanhour University, Damanhour, Egypt

**Keywords:** anti-inflammatory cytokines, COVID-19, pro-inflammatory cytokines, testosterone, TMPRSS2****

## Abstract

COVID-19 is a pandemic disease caused by severe acute respiratory coronavirus 2 (SARS-CoV-2), which leads to pulmonary manifestations like acute lung injury (ALI) and acute respiratory distress syndrome (ARDS). In addition, COVID-19 may cause extra-pulmonary manifestation such as testicular injury. Both high and low levels of testosterone could affect the severity of COVID-19. Herein, there is substantial controversy regarding the potential role of testosterone in SARS-CoV-2 infection and COVID-19 severity. Therefore, the present study aimed to review and elucidate the assorted view of preponderance regarding the beneficial and harmful effects of testosterone in COVID-19. A related literature search in PubMed, Scopus, Web of Science, Google Scholar, and Science Direct was done. All published articles related to the role of testosterone and COVID-19 were included in this mini-review. The beneficial effects of testosterone in COVID-19 are through inhibition of pro-inflammatory cytokines, augmentation of anti-inflammatory cytokines, modulation of the immune response, attenuation of oxidative stress, and endothelial dysfunction. However, its harmful effects in COVID-19 are due to augmentation of transmembrane protease serine 2 (TMPRSS2), which is essential for cleaving and activating SARS-CoV-2 spike protein during acute SARS-CoV-2 infection. Most published studies illustrated that low testosterone levels are linked to COVID-19 severity. A low testosterone level in COVID-19 is mainly due to testicular injury, the primary source of testosterone.

## Introduction

The novel coronavirus disease 19 (nCoV19), commonly known as COVID-19, is an infectious disease caused by severe acute respiratory coronavirus 2 (SARS-CoV-2), leading to acute systemic disturbances, pro-inflammatory activation, hypercytokinemia, cytokine storm, and multi-organ damage (Al-Kuraishy et al., 2020). COVID-19 affects various organs, mainly the respiratory system, presenting with pulmonary manifestations like acute lung injury (ALI) and acute respiratory distress syndrome (ARDS), and extra-pulmonary manifestations like acute cardiac, neurological disorders, pancreatic injury, acute kidney injury, and testicular injury (Al-Kuraishy et al., 2020a; Lugnier et al., 2021). This systemic effect of COVID-19 is due to the wide distribution of angiotensin-converting enzyme 2 (AEC2), a receptor and entry point for SARS-CoV-2 (Al-Kuraishy et al., 2020b). ACE2 receptor is chiefly expressed in the lung alveolar cells type II, proximal renal tubules, and testis primarily in Sertoli and Leydig cells. Binding of SARS-CoV-2 to ACE2 leads to downregulation of these protective receptors with subsequent increment in the level of vasoconstrictors angiotensin II (Ang II) and reduction of vasodilator angiotensin (Ang 1-7) (Ang 1-9) with induction release of pro-inflammatory cytokines (Bank et al., 2021).

Since the World Health Organization (WHO) declaration of this disease as a pandemic and until late July 2021, the total confirmed cases are 194,250,977, with 4,258,789 deaths. The mortality rate ranges from 0.9% to 10.5% in COVID-19 patients without comorbidities than COVID-19 patients with comorbidities, respectively (Anjorin et al., 2021).

It has been reported that male sex is regarded as a risk factor for COVID-19 severity and had worse outcomes, which might occur due to male-specific factors that increase men’s vulnerability to the SARS-CoV-2 infection compared to women (Farghaly and Makboul, 2021). One of the important male sex-specific factors is the anabolic testosterone hormone secreted mainly from testicles and to a lesser extent from the adrenal cortex (Al-Maiahy et al., 2020). Testosterone is also secreted from ovaries in females; however, the total daily testosterone production is approximately 20 times more in males than in females; thus, testosterone serum level is 8 times more in men than in women (Handelsman et al., 2018).

Testosterone serum levels are reduced on average by 2% per year after the age of 40 years, increasing the prevalence of hypogonadism in men following the age of 40 years up to 9.5%, and this prevalence is augmented in several cardiometabolic disorders (Grossmann et al., 2020). Indeed, testosterone deficiency-induced late hypogonadism is regarded as an independent risk factor for various pulmonary disorders and cardio-metabolic disturbances, including hypertension, dyslipidemia, type 2 diabetes mellitus (T2DM), endothelial dysfunction, and coagulopathy (Butanis et al., 2017; Assyov et al., 2020). Therefore, hypogonadism accounts for 53.3% of hospitalized patients with a high mortality rate due to immunosuppression and susceptibility for different viral infections (Pezzaioli et al., 2020).

During COVID-19, SARS-CoV-2 infection may affect the testicles by binding to ACE2 expressed in the Sertoli and Leydig cells, causing infertility and suppressing testosterone production (Abobaker and Raba, 2020). Schroeder et al. (2020) illustrated that low testosterone serum level is linked to SARS-CoV-2 infections and COVID-19 severity in critically ill patients due to reduced immunomodulation antiviral effects of androgen. On the other hand, Wambier et al. (2020) revealed that high testosterone and other androgens serum levels might increase the severity of COVID-19 through augmentation of the expression of transmembrane protease serine 2 (TMPRSS2), which is vital for cleaving and activation of SARS-CoV-2 spike protein during acute SARS-CoV-2 infection.

Therefore, there is substantial controversy regarding the potential role of testosterone and other androgens in SARS-CoV-2 infection and COVID-19 severity. Thus, the present study is aimed to review and elucidate the assorted view of preponderance regarding testosterone’s beneficial and harmful effects in COVID-19.

## Methods and Search Strategy

**Figure 1:**
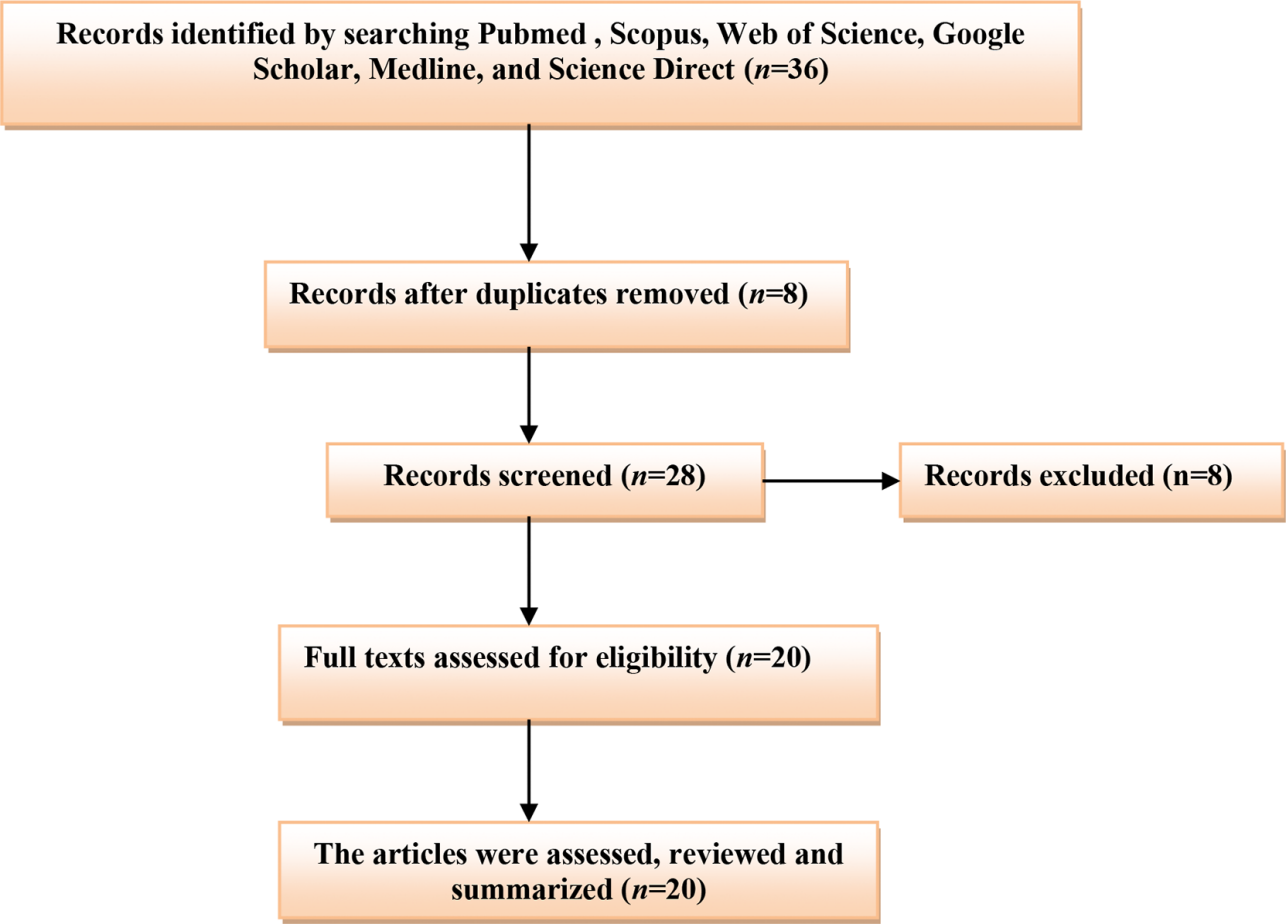
Consort-flow diagram of the present study.

A related literature search in PubMed, Scopus, Web of Science, Google Scholar, and Science Direct was done. All published articles related to the role of testosterone and COVID-19 were included in this mini-review. We search the international database using the medical subject heading (MeSH) to identify the relevant articles published up to 2021. The listed keywords used in this search included [COVID-19 OR SARS-CoV-2] AND [Testosterone OR Androgens], [COVID-19 OR SARS-CoV-2] AND [Hypogonadism OR Androgen sensitivity], [Hypogonadism OR Low testosterone], AND [COVID-19 severity]. The final results were mainly limited to human subjects. All types of published articles with different languages were included, and the final findings were summarized in a mini-review (**Figure 1**).

## The Beneficial Role of Testosterone in COVID-19

It has been reported that testosterone serum level is reduced by aging and cardio-metabolic diseases including T2DM, obesity, dyslipidemia, heart failure, and atherosclerosis, which are common risk factors for the development of COVID-19 severity (Millar et al., 2016; Maddaloni et al., 2020).

Different studies illustrated that testosterone has a protective role in the respiratory system; it improves forced expiratory volume, vital capacity, oxygen consumption, and respiratory muscle contraction (Montano et al., 2014). Marques et al. (2020) illustrated that testosterone replacement therapy in orchiectomized male rats improves oxygenation and attenuates tissue hypoxia and hypercapnia. Thus, low testosterone serum levels in patients with hypogonadism may increase severity of obstructive pulmonary disease (Novković et al., 2019).

### Testosterone and Acute Lung Injury

Testosterone has a crucial pulmo-protective effect through the modulation of lung inflammations, and a reduction of testosterone by aging may predispose the old age for chronic inflammatory pulmonary disorders and viral infections (Keilich et al., 2019). Redente et al. (2011) illustrated that testosterone’s defending role against bleomycin-induced ALI is through inhibition of pro-inflammatory mediated neutrophil alveolitis. It has been observed that testosterone inhibits the production of pro-inflammatory cytokines, including IL-1β, IL-6, and TNF-α, and inflammatory adipocytokines with a cumulative effect on the anti-inflammatory adiponectin (Bianchi, 2019). Wang et al. (2021) revealed that testosterone therapy reduces lung inflammation and fibrosis by inhibiting nuclear respiratory factor 1 (NRF1) and the NF-κB signaling pathway.

Pro-inflammatory cytokines, mainly IL-6, are involved in the pathogenesis of ALI, ARDS, and cytokine storm-induced multi-organ damage in COVID-19 (Sun et al., 2020). Typically, testosterone inhibits the synthesis and release of IL-6 and downregulates the expression of IL-6 receptors (Sun et al., 2020). Thus, IL-6 serum level is augmented in hypogonadism patients that increase their susceptibility for COVID-19 severity (Papadopoulos et al., 2020). Besides, adiponectin, through its anti-inflammatory effects, may reduce SARS-CoV-2-induced pro-inflammatory hypercytokinemia and associated ALI (Messina et al., 2020).

Of note, activation of nod-like receptor pyrin 3 (NLRP3) inflammasome is linked to over-activation of NF-κB signaling-induced inflammatory reactions in COVID-19. Activation of NLRP3 inflammasome is also related with COVID-19 severity and associated complications like ALI and ARDS (van den Berg and Te Velde, 2020). Chen et al. (2020) in their experimental study confirmed that testosterone therapy reduces atherogenesis by suppressing NLRP3 inflammasome. However, Alves et al. (2020) showed that supra-physiological testosterone level leads to oxidative stress-induced endothelial dysfunction and vascular injury through stimulation of NLRP3 inflammasome. Previously, Vignozzi et al. (2012) disclosed that testosterone through its derivative dihydrotestosterone suppresses NF-κB signaling activation and associated pro-inflammatory activations. Therefore, testosterone therapy may improve the clinical outcomes in COVID-19 patients through NLRP3 inflammasome/NF-κB-dependent activation of anti-inflammatory cytokines and suppression of pro-inflammatory cytokines.

A prospective study involving 221 hospitalized men with COVID-19 pneumonia aged > 18 years illustrated that testosterone serum levels are reduced and correlated with COVID-19 severity and mortality (Çayan et al., 2020). Similarly, low baseline total and free testosterone in 31 COVID-19 patients recovered from ARDS in Italy are negatively correlated with inflammatory risk factors (ferritin, CRP procalcitonine, and D-dimer) and linked to COVID-19 severity (Rastrelli et al., 2020). Likewise, an observational study in Germany involving 45 COVID-19 patients revealed that 70% of them have low testosterone at the time of admission with subsequent reduction through ARDS development and admission to the intensive care unit (ICU) (Schroeder et al., 2020). Furthermore, a prospective study composed of 358 men with COVID-19 compared with 92 negative for COVID-19 illustrated that testosterone levels are reduced in COVID-19 patients and linked to poor clinical outcomes (Cinislioglu et al., 2021). In addition, a retrospective study showed that low testosterone level is connected with COVID-19 severity and risk of death (Okçelik, 2021). Low testosterone serum level is associated with reduction of body anti-inflammatory capacity with augmentation of pro-inflammatory axis. High pro-inflammatory cytokines in turn suppress release and action of testosterone in a vicious cycle manner (Bianchi, 2019).

Furthermore, the receptor for advanced glycation end-product (RAGE) is a member of immunoglobulin superfamily protein, which presents in two forms, membrane RAGE (mRAGE) and soluble RAGE (sRAGE) (Papadopoulos et al., 2020). mRAGE has an inflammatory effect through activation of NF-kB, while sRAGE has anti-inflammatory effects through upregulation of ACE2 and anti-inflammatory cytokines (Serveaux-Dancer et al., 2019). RAGE pathway is mainly expressed in lung tissue and linked to the development of acute and chronic lung injuries (Wang et al., 2018). It has been shown that SARS-CoV-2 activates mRAGE at pulmonary alveolar cells, leading to severe inflammatory reactions (Yalcin Kehribar et al., 2020). It has been confirmed that the concentration of sRAGE is reduced with aging, which might explain the susceptibility of old age to COVID-19 (Evens et al., 2020). However, in young and asymptomatic COVID-19 patients, the concentration of sRAGE is high. In severe COVID-19, sRAGE level is significantly reduced, so low sRAGE level is associated with progression of ALI and ARDS (Abbasi-Oshaghi et al., 2021).

Therefore, COVID-19-induced reduction in circulating testosterone may induce ALI due to increase of pro-inflammatory and reduction anti-inflammatory effects.

### Testosterone and Testicular Injury

In COVID-19, SARS-CoV-2 may bind testicle ACE2, leading to Sertoli and Leydig cells’ damage with subsequent inhibition of testicular testosterone synthesis (Illiano et al., 2020). Also, local inflammatory reaction in the testes due to SARS-CoV-2 infection and deregulation of the testicular renin–angiotensin system (RAS) may also impair testicular testosterone synthesis leading to hypogonadism (Yang et al., 2020). Analysis of testicular biopsies in patients with COVID-19 illustrated that the histopathological changes like hypoxic injury and microthrombosis are similar to that observed in COVID-19-induced ALI. However, SARS-CoV-2 was not detected in the injured testes, suggesting oxidative stress; coagulation disorders might mediate this damage as evident in COVID-19 pneumonia (Flaifel et al., 2021).

Therefore, preexistence or SARS-CoV-2-induced hypogonadism may reduce the protective effect of testosterone against SARS-CoV-2 infection, suggesting a link between testicular injury and development of ALI and ARDS in COVID-19 patients (Yang et al., 2020; Zaim et al., 2020). Moreover, high pro-inflammatory cytokines in SARS-CoV-2 infection may induce endothelial dysfunction and coagulopathy, a hallmark in COVID-19. The pro-thrombotic status and risk of thromboembolism are highly aggravated in hypogonadism (Fei et al., 2020). Local testicular thrombosis during SARS-CoV-2 infection is associated with diffuse damage of Leydig and Sertoli cells (Duarte-Neto et al., 2021). However, testosterone supplementation improves endothelial function *via* activation of nitric oxide release, inhibiting platelet activations and pro-thrombotic cascades (Hotta et al., 2019).

These clinical studies illustrated that reduction in the testosterone level is due to testicular injury with a subsequent reduction in the synthesis and release of testosterone from Leydig cells. This simple explanation is not acceptable since testicular injury is not frequently involved during SARS-CoV-2 infections (Schroeder et al., 2020). However, total testosterone may reduce in COVID-19 in the absence of testicular injury, as 90% of COVID-19 patients have a negative test for SARS-CoV-2 in the testes (Yang et al., 2020). A recent study illustrated that hypogonadism is developed in the early phase of COVID-19 due to SARS-CoV-2-induced testicular injury (Dutta and Sengupta, 2021). Higher expression of ACE2 in the testes makes them a potential target for SARS-CoV-2 with subsequent progression of male infertility. Excessive production of reactive oxygen species by SARS-CoV-2 may disrupt sperm function and morphology leading to early- or late-onset infertility (Esteves et al., 2021). Xu et al. (2021) showed that despite testicular injury during acute SARS-CoV-2 infection, male sex hormones remain unchanged even after recovery from COVID-19. Herein, extensive molecular studies are recommended to observe the implication of SARS-CoV-2 infections in reducing testosterone levels in COVID-19 patients. Zhao et al. (2016) illustrated that activation of mRAGE is correlated with inhibition of Leydig cell function with reduction of testosterone biosynthesis. This finding might explain low testosterone levels in patients with severe COVID-19.

In COVID-19, downregulation of lung ACE2 by SARS-CoV-2 is associated with high circulating AngII level, which is linked to development and progression of ALI and ARDS (Zhang et al., 2020). It has been confirmed that AngII inhibits Leydig cell function and testosterone synthesis (Reis and Reis, 2020). Add to these findings, the testes have full RAS, which is involved in the regulation function of Leydig cells and testosterone biosynthesis (Reis and Reis, 2020). Thus, systemic or testicular AngII levels are augmented due to downregulation of ACE2 in COVID-19. Local and circulating AngII activate harmful AT1R on the Leydig cells leading to the inhibition of testosterone biosynthesis (Pascolo et al., 2020). The deregulation of the protective AT2R and Mas receptors within the testes provokes inflammatory cascades that also contribute to Leydig cells’ dysfunction (Aitken, 2020; Pascolo et al., 2020). From the above considerations, AngII might be the potential biomarker linking ALI and testicular injury in patients with severe COVID-19.

### Testosterone and Oxidative Stress

Additional studies illustrated that SARS-CoV-2 infection leads to oxidative stress injury and oxidative storm due to membrane lipid and protein peroxidations (Ntyonga-Pono, 2020). The high neutrophil ratio in SARS-CoV-2 infection is linked to high oxidative stress due to the production of reactive oxygen species (ROS) by neutrophils. These changes provoke a cascade of immuno-biological events that the human body responds to (Ntyonga-Pono, 2020). ROS causes various pathological events related to COVID-19, such as endothelial dysfunction, erythrocyte injury, platelet activation, and thrombosis (Laforge et al., 2020). High ROS in COVID-19 promotes neutrophil extracellular traps (NETs) and induction release of pro-inflammatory cytokines (Laforge et al., 2020). NETs activate NLRP3 inflammasome, NF-κB, and induction of coagulopathy (Schönrich et al., 2020).

It has been reported that oxidative stress inhibits testosterone biosynthesis through activation of mitogen-activated protein kinase p38 (MAPK), which alter the metabolic process and gene expression (Shi and Dansen, 2020). Therefore, severe oxidative stress upregulates the p38MAPK pathway in the Leydig cells causing significant inhibition of testicular testosterone biosynthesis (Han et al., 2018). Recently, Jing et al. (2020) confirmed that oxidized low-density lipoprotein (oxLDL) inhibits testosterone synthesis through induction of p38MAPK pathway in the Leydig cells.

Oxidative stress inhibits Leydig and adrenal cells to synthesize testosterone through upregulation of cyclooxygenase 2 (COX2), induced by the p38MAPK pathway (Martin and Touaibia, 2020). Both p38MAPK pathway and COX2 are activated in COVID-19; Grimes and his colleague (Grimes and Grimes, 2020) illustrated that SARS-CoV-2 might directly or indirectly activate the p38MAPK pathway through downregulation of ACE2 and augmentation of AngII. Besides, activation of pro-inflammatory cytokines in COVID-19 induces upregulation of COX2 (Ong et al., 2020). Zhoa et al. (2019) confirmed that the NF-κB signaling pathway mediates the interaction between the p38MAPK pathway and COX2 in reducing testicular testosterone biosynthesis. In addition, activated p38MAPK provokes blood-testes barrier injury by suppressing testicular spermatogenesis and testosterone biosynthesis (Liu et al., 2018). Therefore, SARS-CoV-2 infection may reduce circulating testosterone and induces hypogonadism through activation of the p38MAPK/COX2 axis.

Into the bargain, testosterone inhibits neutrophil oxidative stress by reducing the production of superoxide anion, inhibition of lipid peroxidation, and improvement of glutathione reductase activity (Marin et al., 2010). In addition, an experimental study revealed that testosterone improves testes antioxidant potential by which it may attenuate oxidative stress-induced testicular injury (Aydilek et al., 2004).

Therefore, testosterone may reduce COVID-19 severity through mitigation of SARS-CoV-2-induced oxidative stress and associated complications.

### Testosterone and Macrophage Function

Moreover, SARS-CoV-2 infection may lead to macrophage activation syndrome (MAS), which is characterized by hemophagocytosis, pancytopenia, coagulopathy, and disseminated intravascular coagulation (DIC). The MAS is developed in different viral infections including SARS-CoV-2 due to imbalanced release of pro-inflammatory cytokines (McGonagle et al., 2021). Of note, testosterone has an important regulatory role on the macrophage, monocyte, and T-cell functions. Testosterone inhibits release of pro-inflammatory and inflammatory cytokines from immune cells (Bereshchenko et al., 2018). Testosterone therapy was shown to prohibit release of pro-inflammatory cytokines from monocytes mainly in hypogonadal men compared with eugonadal one (Bianchi, 2019). In addition, testosterone decreases the expression and sensitivity of macrophage TLR4 for its ligand (Rettew et al., 2008). Of interest, TLR4 mediates early immunological interaction of SARS-CoV-2 with macrophage and other immune cells (Aboudounya and Heads, 2021). Therefore, testosterone therapy in COVID-19 patients may interrupt macrophage activation, exaggerated immune response, and development of MAS.

Furthermore, testicular macrophages (TMs) have immunoregulatory and immunotolerant functions as well as control of testicular steroidogenesis and spermatogenesis (Meinhardt et al., 2018). During sepsis and pathogen invasion, the classical type macrophage (M1) is activated and induces release of local pro-inflammatory cytokines. These cytokines impair spermatogenesis with significant inhibition of testicular steroidogenesis. The alternative type macrophage (M2) has local anti-inflammatory action supporting spermatogenesis and release of testosterone from Leydig cells (Chen et al., 2018; Meinhardt et al., 2018).

In SARS-CoV-2 infection, macrophage polarization is toward M1 phenotype resulting in testicular injury with impairment of testicular steroidogenesis and spermatogenesis (Lv et al., 2021). Becerra-Diaz et al. (2018) illustrated that testosterone and other androgens through macrophage androgenic receptor (AR) enhance M2 polarization with domination of macrophage anti-inflammatory effect. Taken together, testosterone modulates macrophage functions in general and more specifically TMs, by which it reduces the harmful effects of SARS-CoV-2 infection on the testes.

### Testosterone Versus Estrogen in Men

In general, women have a robust immune system as compared to men due to the protective effect of estrogen against immunological dysregulation during different viral infections (Priyanka and Nair, 2020). It has been shown that estrogen has complex immunomodulating effects, and its effect on the inflammatory milieu in COVID-19 has been suggested (Ma et al., 2021). High estrogen serum level in premenopausal women might be a protective factor against COVID-19 severity, though older post-menopausal women are of high risk for development of COVID-19 severity compared to elderly men (Ciarambino et al., 2021). Nevertheless, reduction of estrogen level in later life in women does not appear to play a harmful role regarding COVID-19 severity in elderly women (Papadopoulos et al., 2021). In elderly men, there is significant reduction of testosterone with elevation of estrogen level due to increasing aromatization of adrenal and testicular androgens (Jardí et al., 2018). However, during sepsis in men, there is a noteworthy reduction of testosterone level with parallel increase of estrogen that reflects negative outcomes in septic men (Bech et al., 2020).

Thus, administration of estrogen in men with COVID-19 may offer a potential protective effect against COVID-19 severity (Suba, 2020). Bukowska et al. (2017) confirmed from experimental data that estrogen is able to regulate expression of the ACE/ACE2 axis, which is highly distorted in COVID-19. Also, estrogen inhibits propagation of cytokine storm and can activate B cells for antibody production. Besides, estrogen reduces expression of TMPRSS2, thereby reducing the entry of SARS-CoV-2 to the susceptible cells (Bennink et al., 2021). So, estrogen treatment is suggested to be an effective treatment against COVID-19 (Bennink et al., 2021).

**Figure 2:**
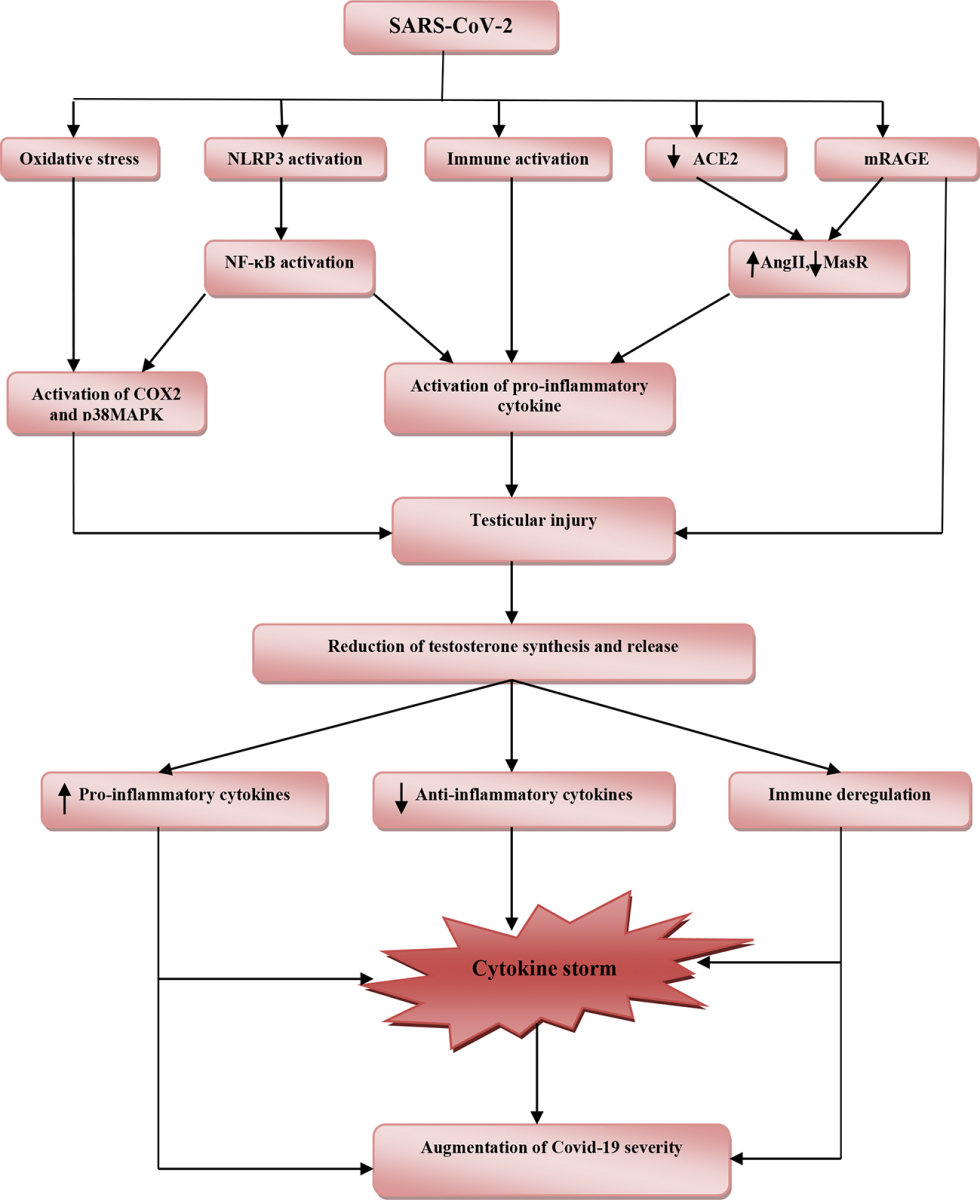
The potential role of SARS-CoV-2 infection in the reduction of testosterone and associated COVID-19 severity. SARS-CoV-2 induces oxidative stress, activation of nod-like receptor pyrin-3 (NLRP3) inflammasome and abnormal immune activation, downregulation of angiotensin converting enzyme 2 (ACE2), and activation of receptor for advanced glycation end-product (mRAGE). Downregulation of ACE2 with activation of mRAGE increases angiotensin II (AngII) and reduces Mas receptor (MasR). Activation of NLRP3 inflammasome triggers release of nuclear factor kappa B (NF-κB) and together with cyclooxegenase-2 (COX-2) and p38 mitogen-activated protein kinase (p38MAPK) stimulate release of pro-inflammatory cytokines, which cause testicular injury. These pathophysiological changes reduce production and release of testosterone from injured testes. Reduction in the level of testosterone provokes releases of pro-inflammatory cytokines and reduces anti-inflammatory cytokines with immune deregulation. These changes lead to induction of cytokine storm with consequent augmentation of COVID-19 severity.

These findings highlighted the potential protective effects of testosterone against SARS-CoV-2 infection (**Table 1**). However, reduction of total testosterone level in COVID-19 is due to complex interactions between SARS-CoV-2 with oxidative stress, pro-inflammatory cytokines, and systemic and local RAS (**Figure 2**).

**Table 1:** Beneficial effects of testosterone in COVID-19.

References	Study type	Findings
Margue et al. (Marques et al., 2020)	Experimental study	Testosterone improves oxygenation and attenuates tissue hypoxia.
Wang et al. (2021)	Experimental study	Testosterone therapy reduces lung inflammation and fibrosis.
Bianch (Sun et al., 2020)	Systematic review	Testosterone inhibits the synthesis and release of IL-6.
Chen et al. (2020)	Experimental study	Testosterone therapy inhibits NLRP3 inflammasome.
Vignozzi et al. (2012)	Prospective study	Testosterone suppresses NF-κB signaling.
Cayan et al. (Çayan et al., 2020)	Cohort study	Testosterone serum levels are reduced and correlated with COVID-19 severity and mortality.
Rastrelli et al. (2020)	Cohort study	Testosterone serum level is negatively correlated with inflammatory risk factors
Schroder et al. (Rastrelli et al., 2020)	Cohort study	70% of COVID-19 patients have low testosterone at the time of admission.
Cinislioglu et al. (2021)	Prospective study	Testosterone levels are reduced in COVID-19 patients and linked to poor clinical outcomes.
Okcelik et al. (Okçelik, 2021)	Retrospective study	Low testosterone level is connected with COVID-19 severity and risk of death.
Hota et al. (Hotta et al., 2019)	Systematic review	Testosterone supplementation improves endothelial function.
Marin et al. (2010)	*In vitro* study	Testosterone inhibits oxidative stress.
Bereshchenko et al. (2018)	Systematic review	Testosterone inhibits release of pro-inflammatory and inflammatory cytokines from immune cells.

## Harmful Role of Testosterone in COVID-19

### Testosterone and TMPRSS2 in COVID-19

Various studies illustrated that men’s higher predisposition to develop severe and serious COVID-19 complications is related to sex hormones, mainly testosterone and sociocultural factors (Lipsky and Hung, 2020). It has been confirmed that TMPRSS2 is required for proteolytic activation and priming of SARS-CoV-2 spike protein to bind ACE2 (Rahman et al., 2020). The expression of the TMPRSS2 gene is promoted by testosterone hormone, which might explain the severity of COVID-19 in men due to facilitating the entry of SARS-CoV-2 (Stopsack et al., 2020). TMPRSS2 is a cellular enzyme encoded by the human TMPRSS2 gene involved in prostatic cancer (Mehra et al., 2007) and cleaving of hemagglutinin viral antigen and infectivity of HIN1 and H7N9 influenza virus (Cheng et al., 2015). The TMPRSS2 gene is expressed in different tissues including lung and testes (Shen et al., 2020).

In addition to the androgen, nicotine smoking increases the expression of the TMPRSS2 gene, which might explain the severity of COVID-19 severity in nicotine smoker patients (Voinsky and Gurwitz, 2020). However, various studies reported the protective effect of nicotine smoking against SARS-CoV-2 due to different mechanisms, including upregulation of lung ACE2, anti-inflammatory, and immunosuppressive effects through activation of nicotinic acetylcholine receptor type 7 alpha (nAChR7α) on the macrophage (Farsalinos et al., 2020). Likewise, Donlan et al. (2020) observed that the expression of the TMPRSS2 gene is activated by IL-13, a highly expressed cytokine in COVID-19 and regarded as a predictive factor for mechanical ventilation independent of gender, age, and comorbidities. Besides, TMPRSS2 is highly co-expressed with furin, cathepsin L and B, CD209, and CD147 in men only, while co-expression with ACE2 is similar in both sexes (Piva et al., 2021). Certainly, TMPRSS2 co-expression with CD147 is important since CD147 is regarded as an entry point for SARS-CoV-2 (Radzikowska et al., 2020). Furthermore, Cao et al. (2017) in their experimental study confirmed that testosterone therapy increases the expression of CD147.

Therefore, overexpression of TMPRSS2 by androgen may implicate the testosterone in the pathogenesis of SARS-CoV-2 infection and COVID-19 severity. Thereby, TMPRSS2 inhibitors such as bromhexine, aprotinin, camostat, and nafamostat are useful in managing COVID-19 through attenuation of TMPRSS2-dependent lung inflammation, coagulopathy, and development of ARDS (Azimi, 2020; Breining et al., 2020). Indeed, the population-based study of Montopoli et al. (2020) that involved 9,280 COVID-19 patients with or without prostatic cancer illustrated that patients receiving androgen deprivation therapy (ADT) are at a lower risk for COVID-19-related complications compared to patients who did not receive ADT. This finding suggests that the anti-androgen agents reduce testosterone’s activation role on the expression of TMPRSS2, and thus high testosterone level may increase COVID-19 severity. Adamowicz et al. (2020) showed that high dihydrotestosterone level is linked to poor pulmonary outcomes in COVID-19 patients, though use of 5-α reductase inhibitors may aggravate COVID-19 severity due to disturbance of intra-pulmonary androgen metabolism. However, McCoy et al. (2020) showed that using 5-α reductase inhibitors is associated with good clinical outcomes in COVID-19 patients.

Moreover, different therapeutic modalities such as dexamethasone, nitric oxide, and chloroquine, which are effective in managing COVID-19, are reported to have anti-androgenic effects and suppression of TMPRSS2 (Cronauer et al., 2007; Guo et al., 2018; Chi et al., 2020). Taken together, based on the current findings, testosterone is implicated in the facilitation of SARS-CoV-2 infection through upregulation of TMPRSS2 and androgen receptor (AR) activation.

### Androgen Sensitivity and COVID-19

The role of androgen sensitivity and polymorphism in COVID-19 is explained by different studies. It has been reported that a low mortality rate in pre-pubescent compared to the high mortality rate in adult men during the COVID-19 pandemic is due to low androgen sensitivity (Wambier et al., 2020). In addition, men with androgenic alopecia and women with polycystic ovary syndrome are at a higher risk for SARS-CoV-2 infection and COVID-19 severity due to higher androgen sensitivity. Therefore, the higher mortality rate for COVID-19 in the African American population is related to the polymorphism and higher sensitivity of androgenic receptors (Goren et al., 2020).

It has been known that the polyglutamine (poly-Q) tract of the AR affects the physiological response of circulating testosterone (Callewaert et al., 2003). Longer poly-Q of AR reduces the sensitivity to testosterone and is associated with high testosterone serum level because of impairment of negative feedback inhibition (Mohamad et al., 2018). In addition, longer poly-Q of AR is linked to activation of pro-inflammatory axis (Pierotti et al., 2010), although AR with short poly-Q has protective and anti-inflammatory roles in COVID-19 regardless of testosterone serum levels (Baldassarri et al., 2021). Therefore, testosterone may have bidirectional effects depending on the underlying length of AR poly-Q tract.

The distribution of poly-Q allele differs among diverse populations: longer in Asians, medium in Caucasians, and shorter in Africans (Ackerman et al., 2012). This might explain the high mortality in the first wave of SARS-CoV-2 infection in both China and Italy (Pereira et al., 2020). Of interest, African populations are more prone to the SARS-CoV-2 infection due to higher sensitivity of AR and higher expression of the TMPRSS2 gene (de Lusignan et al., 2020).

Therefore, AR sensitivity and length of poly-Q tract of AR seem to be more important than testosterone level in the prediction of COVID-19 severity. Besides, testosterone therapy in patients with COVID-19 may improve or worsen the clinical outcomes depending on patient AR sensitivity (Mukherjee and Pahan, 2021).

### Immunological Effects of Testosterone in COVID-19

It has been reported that both adaptive and innate immune systems are crucial for contrasting viral infections and enhancing viral clearance and tissue repair (Kikkert, 2020). Giagulli et al. (2021) illustrated that circulating testosterone has immunosuppressive effects by inhibiting B and T cells, impairing immune response and immunoglobulin generations in different viral infections (Ghosh and Klein, 2017). In COVID-19, natural killer, B, and T cells are reduced; specifically reducing CD8 T cell is regarded as an independent predictor for severe COVID-19-related complications (Wang et al., 2020). Kissick et al. (2014) revealed that testosterone inhibits differentiation of CD4 T cells, providing a basis for targeting testosterone and other androgenic receptors to mitigate CD4 T-cell response in various forms of autoimmune disorders.

Several lines of evidence from various studies point to the immunosuppressive potential role of testosterone on various components of the immune system (Gubbels Bupp and Jorgensen, 2018), although the basic molecular mechanism is still not elucidated. Testosterone mediated downregulation of systemic immune response through cell-type-specific effects in many immunological disorders (LaVere et al., 2021). The precise immunological effects of testosterone and other androgens are through inhibition of antibody response to the viral infections and vaccines, suppression of macrophages and dendritic cells, promotion of immunological tolerance *via* activation of regulatory T cells, and inhibition of functions and developments of B and T cells (Trigunaite et al., 2015). Regarding these considerations, men are more vulnerable for COVID-19 severity as compared with women due to the immunosuppressive effects of testosterone (Bwire, 2020). Testosterone enhances both secretion and production of Th1-to-Th2 cytokine ratio *via* stimulated T cells and reduces humoral response and B-cell proliferation (Roved et al., 2017).

Moreover, the lysophosphatidyl serine receptor (GPR174) encoded by X-chromosome gene is highly expressed on B and T cells in women compared with men (Barnes et al., 2015). GPR174 regulates and controls the release of pro-inflammatory cytokines, B-cell migration, and macrophage polarization in septic shock and in response to chemokines (Qiu et al., 2019). These verdicts and results highlighted testosterone’s potential immunosuppressive effect in the progression of SARS-CoV-2 infection and COVID-19 severity (Salonia et al., 2021). Therefore, ADT might be a therapeutic opportunity against COVID-19 by reversal of immunosuppression status (Montopoli et al., 2020).

### Metabolic Effects of Testosterone in COVID-19

Testosterone has a permissive effect for circulating AngII by expressing AT1R and downregulation of ATR2 with a higher ATR1R/AT2R ratio. However, castration reverses this ratio (Mishra et al., 2019). High circulating AngII and ATR1R expression are linked to ALI and ARDS development in COVID-19 (Wu et al., 2020). Besides, a high AngII level induces testicular injury and cell apoptosis with the reduction of Leydig cells for the synthesis of testosterone (Wang et al., 2017). Thus, in SARS-CoV-2 infection, there is a vicious cycle conflict in the interaction between testosterone and AngII concerning the lung–testis axis in severe COVID-19.

To date, dipeptidyl peptidase 4 (DPP4), which is highly expressed in different tissues, mainly in lung type II alveolar cells, is regarded as an entry point for SARS-CoV-2 and is associated with poor clinical outcomes in COVID-19 patients (Solerte et al., 2020). Blauschmidt et al. (2017) observed that testosterone upregulates the expression of DPP4 receptors in women with polycystic ovary syndrome. DPP4 inhibitors effectively manage COVID-19 through modulation of the anti-inflammatory/pro-inflammatory axis (Mirani et al., 2020). Thus, testosterone may increase COVID-19 severity through the DPP4/CD26 pathway; however, there is no study related to DPP4/CD26 and testosterone in SARS-CoV-2 infection.

**Figure 3:**
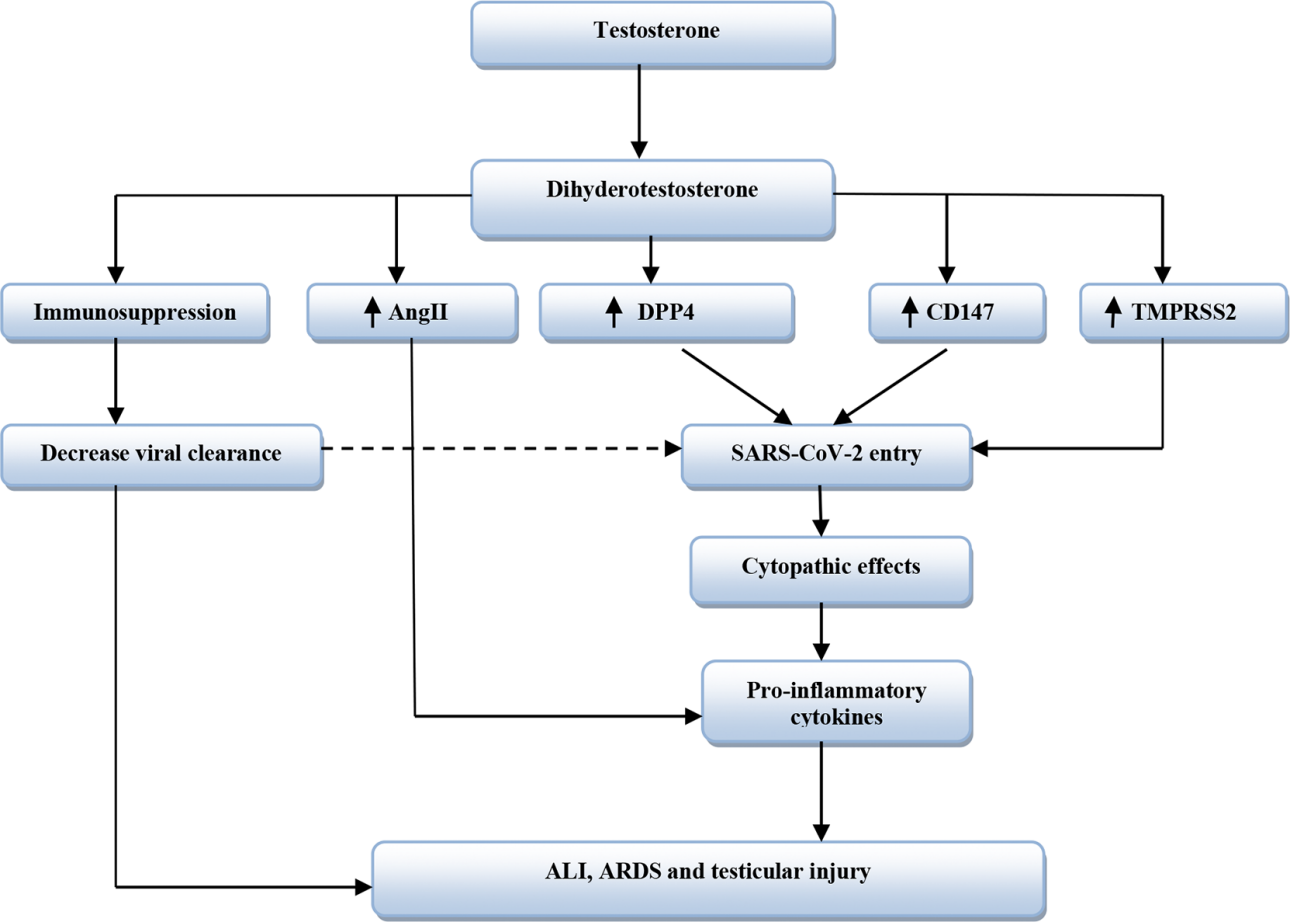
The harmful effects of testosterone in SARS-CoV-2 infection. Dihydrotestosterone from testosterone leads to immunosuppression and increases expression of Dipeptidyl peptidase 4 (DPP4), cluster differentiation 147 (CD147), and Transmembrane protease serine 2 (TMPRSS2). Also, Dihydrotestosterone increases activity of Angiotensin II (AngII). CD147 and DPP4 increase entry of SARS-CoV-2, while TMPRSS2 facilitates SARS-CoV-2 entry. Immunosuppression reduces viral clearance that increases SARS-CoV-2 entry. These changes lead to cytopathic effects with release of pro-inflammatory cytokines with subsequent development of acute lung injury (ALI), acute respiratory distress syndrome (ARDS), and testicular injury.

Moreover, obesity is associated with low circulating testosterone due to aromatization of testosterone to estrogen by adipose tissue and abnormal hypothalamic–pituitary axis (Haring et al., 2010). Obesity is regarded as an independent risk factor for COVID-19 severity despite low testosterone levels (Yang et al., 2021), although ample evidence from experimental, preclinical, and clinical studies revealed that low testosterone level promotes development of obesity (Fui et al., 2014). Testosterone improves catecholamine-induced lipolysis and inhibits uptake of triglyceride by suppressing the activity of adipose tissue lipoprotein lipase (Grossmann, 2011). It has been reported that patients with prostatic cancer on ADT had increased fat mass and visceral adipose tissue by about 22% within 6 months of established therapy (Hamilton et al., 2011). Likewise, experimental hypogonadism in young men induces obesity within 10 weeks (Mauras et al., 1998). Therefore, low testosterone-induced obesity may aggravate the clinical course of COVID-19 severity. Sarver and Wong (2021) showed that obesity increases the expression of TMPRSS2 and DPP4 with alteration of the ACE/ACE2 ratio. Thereby, obesity may increase the risk of SARS-CoV-2 infection and abnormal immune response by underlying high pro-inflammatory cytokines (Seidu et al., 2021). Therefore, testosterone’s harmful effects in COVID-19 are related to TMPRSS2, ATR1, CD147, DPP4, and AngII expression that are mutually interrelated in facilitating SARS-CoV-2 entry and associated inflammatory reactions (**Table 2** and **Figure 3**).

**Table 2:** Harmful effects of testosterone in COVID-19.

References	Study type	Findings
Stopsack et al. (2020)	Systematic review	Testosterone promotes expression of the TMPRSS2.
Cao et al. (2017)	Experimental study	Testosterone therapy increases the expression of CD147.
Montopoli et al. (2020)	Population-based study	Androgen deprivation therapy reduces COVID-19 severity.
Adamowicz et al. (2020)	Observational study	High dihydrotestosterone level is linked to poor pulmonary outcomes in COVID-19 patients.
McCoy et al. (2020)	Observational study	5-α reductase inhibitors is associated with good clinical outcomes in COVID-19 patients.
Wambier et al. (2020)	Observational study	Low androgen sensitivity is linked to low COVID-19 mortality.
Wang et al. (2020); Bwire (2020)	*In vitro* and review studies	Circulating testosterone has immunosuppressive effects.
Mishra et al. (2019)	Experimental study	Testosterone has a permissive effect for circulating AngII by expressing AT1R and downregulation of ATR2 with a higher ATR1R/AT2R ratio.
Blauschmidt et al. (2017)	Case-series study	Testosterone upregulates the expression of DPP4 receptors.

## Conclusion

Testosterone hormone has diverse immunological effects; it reduces B- and T-cell activity with a noteworthy inhibitory effect on macrophage and monocyte activities. Therefore, testosterone has an immunosuppressive effect subjecting male sex for various viral infections including SARS-CoV-2. In the COVID-19 era, different reports, retrospective, and small sample size prospective studies revealed that testosterone serum might correlate with COVID-19 severity and poor clinical outcomes. However, other studies illustrated that testosterone has a protective effect against COVID-19 severity through inhibition of inflammatory signaling pathways including NF-κB, NLRP3 inflammasomes, and p38MAPK. Also, testosterone attenuates oxidative stress-induced endothelial dysfunction and associated coagulopathy, a hallmark of COVID-19.

In the present review, depending on the recent published studies, we divided testosterone effects into beneficial and harmful effects. The beneficial effect of testosterone in COVID-19 is mediated through modulation of the pro-inflammatory/anti-inflammatory axis with inhibition of SARS-CoV-2-induced oxidative stress. Besides, testosterone attenuates development of ALI and ARDS in SARS-CoV-2 and other respiratory viral infections. The harmful effect of testosterone in COVID-19 is mediated by different unidentified mechanisms, although increased expression of TMPRSS2, DPP4, and CD147 by testosterone might be the potential mechanism. These receptors together with TMPRSS2 facilitate entry of SARS-CoV-2 to the affected cells with subsequent cytopathic effects and release of pro-inflammatory cytokines. Moreover, an increase in androgen sensitivity due to polymorphism of androgenic receptors might be a more important mechanism in the prediction of COVID-19 severity than testosterone serum levels.

On the other hand, a low testosterone serum level in COVID-19 patients might be due to direct testicular injury by SARS-CoV-2 or indirectly by the high level of pro-inflammatory cytokines. In addition, SARS-CoV-2-induced oxidative stress may affect testosterone metabolism and action. We suggest that disturbance of the hypothalamic–pituitary–gonadal axis by SARS-CoV-2 infection and associated inflammatory disorders could be the possible mechanism for low testosterone in COVID-19.

However, the assorted view of preponderance showed that low testosterone level is linked to COVID-19 severity. In addition, high inflammatory and oxidative stress burden with downregulation of ACE2 in SARS-CoV-2 infection may lead to testicular injury and reduction of testosterone biosynthesis. Despite these findings, the present study cannot conclude the beneficial or harmful effects of testosterone in COVID-19. Clinical trials and large-scale prospective studies are warranted to confirm the potential associations in this regard.

## Author Contributions

All authors contributed to the article and approved the submitted version.

## Funding

The publication of this study has been supported by the Pnoi Lab – Industrial & Laboratory Equipment.

## Conflict of Interest

The authors declare that the research was conducted in the absence of any commercial or financial relationships that could be construed as a potential conflict of interest.

## Publisher’s Note

All claims expressed in this article are solely those of the authors and do not necessarily represent those of their affiliated organizations, or those of the publisher, the editors and the reviewers. Any product that may be evaluated in this article, or claim that may be made by its manufacturer, is not guaranteed or endorsed by the publisher.
